# Positive association between hypertension and urinary bladder cancer: epidemiologic evidence involving 79,236 propensity score-matched individuals

**DOI:** 10.1080/03009734.2018.1473534

**Published:** 2018-06-18

**Authors:** Victor C. Kok, Han-Wei Zhang, Chin-Teng Lin, Shih-Chung Huang, Ming-Feng Wu

**Affiliations:** a Department of Internal Medicine, Division of Medical Oncology, Kuang Tien General Hospital, Taichung, Taiwan (ROC); b Disease Informatics Research Unit, Asia University Taiwan, Taichung, Taiwan (ROC); c Institute of Population Health Sciences, National Health Research Institutes, Zhunan, Taiwan (ROC); d PhD Program for Aging, China Medical University, Taichung, Taiwan (ROC); e Institute of Electrical Control Engineering, Department of Electrical and Computer Engineering, National Chiao Tung University, Hsinchu, Taiwan (ROC); f Brain Research Center, National Chiao Tung University, Hsinchu, Taiwan (ROC); g Centre for Artificial Intelligence, School of Software, Faculty of Engineering & IT, University of Technology, Sydney, Australia (ROC); h Division of Cardiology, Department of Internal Medicine, Kuang Tien General Hospital, Taichung, Taiwan (ROC); i Department of Medical Intensive Care Unit, Kuang Tien General Hospital, Taichung, Taiwan (ROC)

**Keywords:** Essential hypertension, population-based cohort study, propensity analysis, urinary bladder cancer

## Abstract

**Introduction:**

We hypothesized that hypertensive patients harbor a higher risk of urinary bladder (UB) cancer.

**Material and methods:**

We performed a population-based cohort study on adults using a National Health Insurance Research Database (NHIRD) dataset. Hypertension and comparison non-hypertensive (COMP) groups comprising 39,618 patients each were propensity score-matched by age, sex, index date, and medical comorbidities. The outcome was incident UB cancer validated using procedure codes. We constructed multivariable Cox models to derive adjusted hazard ratios (aHRs) and 95% confidence intervals (CIs). Cumulative incidence was compared using a log-rank test.

**Results:**

During a total follow-up duration of 380,525 and 372,020 person-years in the hypertension and COMP groups, 248 and 186 patients developed UB cancer, respectively, representing a 32% increase in the risk (aHR, 1.32; 95% CI, 1.09–1.60). Hypertensive women harbored a significantly increased risk of UB cancer (aHR, 1.55; 95% CI, 1.12–2.13) compared with non-hypertensive women, whereas men with hypertension had a statistically non-significant increased risk (aHR, 1.22; 95% CI, 0.96–1.55). The sensitivity analysis demonstrated that the increased risk was sustained throughout different follow-up durations for the entire cohort; a statistical increase in the risk was also noted among hypertensive men.

**Conclusion:**

This nationwide population-based propensity score-matched cohort study supports a positive association between hypertension and subsequent UB cancer development.

## Introduction

A prospective, population-based cohort study in people aged over 45 conducted in the United States demonstrates that after a median follow-up of nearly five years, the incidence rate of hypertension per 1,000 person-years is 84.9 for Blacks, 65.7 for Hispanics, 56.8 for Whites, and 52.2 for Chinese (1). Hypertension, as a global public health problem, has also been known to link with multiple medical conditions, in which the association between hypertension and cancer is receiving increasing attention (2–5).

Previous studies have generated inconclusive results on the association between hypertension and urinary bladder cancer (6–8). Urinary bladder (UB) cancer is the sixth most common cancer in the United States, primarily affecting individuals older than 65 years (9). The rationale of the association between hypertension and UB cancer includes the following: hypertension is one component of the metabolic syndrome which has been shown to associate with subsequent cancer development (7); hypertension may increase the risk of developing breast cancer by blocking and subsequently modifying apoptosis, thereby affecting the regulation of cell turnover (2). There is convincing data from animal studies supporting a causative role for oxidative stress in the pathogenesis of hypertension (10), while oxidative stress has been known as a causative factor in the development of cancer.

Given that both hypertension and urinary bladder cancer are major health problems and the unsolved issue of the linkage between them, there is a rationale for examining whether patients with hypertension harbor an increased risk of developing UB cancer. To answer this research question, we designed a nationwide population-based cohort study using a National Health Insurance research dataset to determine whether patients with essential hypertension had an increased risk of developing UB cancer.

## Patients and methods

### Dataset

We conducted a population-based cohort study by retrieving data of all individuals from the Longitudinal Health Insurance Dataset 2000 (LHID2000). The LHID2000 includes the registration files and claims data of ambulatory care expenditures or inpatient expenditures for 1,000,000 individuals systematically and randomly sampled from the year 2000 registry of beneficiaries of the National Health Insurance Research Database (NHIRD). The NHIRD comprises detailed healthcare information from >23 million enrollees, representing >99% of the entire population in Taiwan. The high accuracy of NHIRD records has been validated, and it appears to be a valid resource for population research in cardiovascular diseases (11,12). The authors of the current research are familiar with epidemiologic research using the NHIRD dataset to derive population-based answers for clinical research questions (13–18). This study has received an exemption from a full review from the accredited institutional review board of Kuang Tien General Hospital, Taichung, Taiwan (certificate number KTGH-10629). Furthermore, this study has been registered in the Research Registry (unique identification number 2667).

### Study design

We designed a nationwide population-based prospective study design using historical data and applied propensity score matching (19) to assemble a comparison group, using a longitudinal healthcare claims-based dataset. The alternate hypothesis was that adult patients with hypertension harbored higher risk of UB cancer at follow-up.

### Study cohort assembly

**Figure 1.**
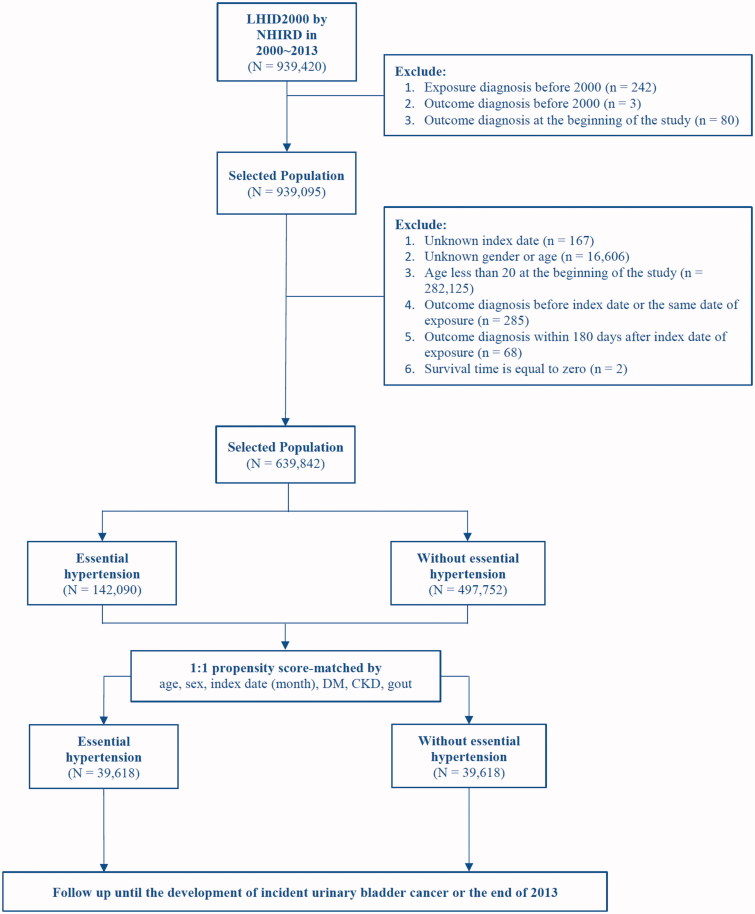
Consort diagram of the study flow.

The LHID2000 dataset contains medical claims data for 939,420 enrollees from the years 2000 to 2013. After excluding individuals with the exposure (hypertension) diagnosis (*n* = 242), individuals with the outcome (UB cancer) diagnosis (*n* = 3) before 2000, and individuals with the outcome diagnosis at the beginning of the study (*n* = 80), 939,095 individuals were sampled. Next, after excluding individuals with an unknown index date (*n* = 167), unknown gender or age (*n* = 16,606), age <20 years at the beginning of the study (*n* = 282,125), outcome diagnosis received before the index date or on the same date as the exposure (*n* = 285), outcome diagnosis received within 180 days after the index date of exposure (*n* = 68), and a survival time of zero (*n* = 2), we recruited a sample population of 639,842 individuals. Among the sample population, 142,090 patients were diagnosed with essential hypertension. During the study period, hypertension was defined as office systolic/diastolic blood pressure ≥140/90 mmHg or ≥130/80 mmHg when antihypertensive treatment was initiated for high-risk patients, such as those with diabetes, chronic kidney disease, stroke, established coronary heart disease, and coronary heart disease equivalents (carotid artery disease, peripheral arterial disease, and abdominal aortic aneurysm) (20). Subsequently, we performed 1-to-1 propensity score matching (PSM) according to six patient characteristics—age, sex, index date (month), diabetes mellitus, chronic kidney disease, and gout—to create one exposure group and one comparison group, each consisting of 39,618 patients (Table 1; Figure 1).

**Table 1.** Baseline characteristics of the essential hypertension group versus the non-hypertensive comparison group before and after propensity score matching.

Characteristics	Before propensity score matching					After propensity score matching			
	Hypertensive group (*n* = 142,090)	Non-hypertensive group (*n* = 497,752)	Standardized mean difference	*P* value		Hypertensive group (*n* = 39,618)	Non-hypertensive group (*n* = 39,618)	Standardized mean difference	*P* value
Age			0.9683	<0.001^b^				0.0000	0.999^b^
Mean (Median)	54.22 (52.92)	39.92 (37.00)				56.35 (55.75)	56.35 (55.75)		
IQR	43.75 (65.08)	28.17 (47.83)				46.17 (67.17)	46.17 (67.17)		
Gender, *n* (%)			0.0179	<0.001^a^				0.0000	0.999^a^
Female	71539 (50.3)	245237 (49.3)				19218 (48.5)	19218 (48.5)		
Male	70551 (49.7)	252515 (50.7)				20400 (51.5)	20400 (51.5)		
Diabetes mellitus			0.6624	<0.001^a^				0.0000	0.999^a^
No	91797 (64.6)	454558 (91.3)				28105 (70.9)	28105 (70.9)		
Yes	50293 (35.4)	43194 (8.7)				11513 (29.1)	11513 (29.1)		
Chronic kidney disease			0.3881	<0.001^a^				0.0000	0.999^a^
No	126738 (89.2)	488143 (98.1)				37289 (94.1)	37289 (94.1)		
Yes	15352 (10.8)	9609 (1.9)				2329 (5.9)	2329 (5.9)		
Gout			0.4508	<0.001^a^				0.0000	0.999^a^
No	111768 (78.7)	468215 (94.1)				33559 (84.7)	33559 (84.7)		
Yes	30322 (21.3)	29537 (5.9)				6059 (15.3)	6059 (15.3)		
Chronic cystitis			0.0441	<0.001^a^				0.0043	0.542^a^
No	141385 (99.5)	496673 (99.8)				39449 (99.6)	39460 (99.6)		
Yes	705 (0.5)	1079 (0.2)				169 (0.4)	158 (0.4)		
Spinal cord injury			0.0694	<0.001^a^				0.0138	0.053^a^
No	140575 (98.9)	495587 (99.6)				39252 (99.1)	39198 (98.9)		
Yes	1515 (1.1)	2165 (0.4)				366 (0.9)	420 (1.1)		
Pesticide exposure			0.0205	<0.001^a^				0.0037	0.598^a^
No	141846 (99.8)	497301 (99.9)				39550 (99.8)	39556 (99.8)		
Yes	244 (0.2)	451 (0.1)				68 (0.2)	62 (0.2)		
Alcohol-use disorders			0.0698	<0.001^a^				0.0107	0.132^a^
No	138684 (97.6)	491032 (98.6)				38835 (98.0)	38893 (98.2)		
Yes	3406 (2.4)	6720 (1.4)				783 (2.0)	725 (1.8)		
Smoking-related diagnoses			0.2437	<0.001^a^				0.0203	0.004^a^
No	125388 (88.2)	474353 (95.3)				34978 (88.3)	35234 (88.9)		
Yes	16702 (11.8)	23399 (4.7)				4640 (11.7)	4384 (11.1)		
Morbid obesity			0.1341	<0.001^a^				0.0833	<0.001^a^
No	139673 (98.3)	495846 (99.6)				39081 (98.6)	39401 (99.5)		
Yes	2417 (1.7)	1906 (0.4)				537 (1.4)	217 (0.5)		
Chronic liver disease			0.3254	<0.001^a^				0.1318	<0.001^a^
No	112529 (79.2)	455061 (91.4)				31900 (80.5)	33857 (85.5)		
Yes	29561 (20.8)	42691 (8.6)				7718 (19.5)	5761 (14.5)		

Propensity score uses nearest-neighbor method matching, by age, sex, index date, diabetes mellitus, chronic kidney disease, and gout.

Standardized mean difference is a companion to the book by Mark W. Lipsey and David B. Wilson, *Practical Meta-analysis*, published in 2001 by Sage. An older Excel-based version of the calculator can be found at http://mason.gmu.edu/∼dwilsonb/ma.html.

ICD-9-CM code of comorbidities: Essential hypertension (401–405), urinary bladder cancer (188, 233.7), smoking-related diagnoses (305.1, 491.0, 491.2, 492.8, 496, 523.6, 989.84, V15.82, 649.0), alcohol-use disorders (265.2, 291, 303, 305.0, 357.5, 425.5, 535.3, 571.0, 571.1, 571.2, 571.3, 980.0, V11.3), morbid obesity (278, 646.1, 649.1, 649.2, V45.86, V65.3, V77.8), chronic cystitis (595.1, 595.2), spinal cord injury (806, 952, 336.1), chronic liver disease (571, 572.2–572.9), diabetes mellitus (249, 250, 648.8, 648.0), gout (274), chronic kidney disease (403, 404, 582.9, 585, 646.2, 792.5, 996.1, 999), pesticide exposure (989.1, 989.2, 989.3, 989.4).

^a^*P* value comes from chi-square test.

^b^*P* value comes from two-sample *t* test.

### Outcome measures

The outcome of this research was incident UB cancer defined as a diagnosis made at least three times by a physician or urologist in ambulatory settings or at least once with the same diagnosis made in the discharge diagnoses, using *International Classification of Diseases, Ninth Edition, with Clinical Modification* (ICD-9 CM) codes. In this study, UB cancer included any histopathological type of malignancy involving the UB, which included urothelial carcinoma, adenocarcinoma, squamous cell carcinoma, and sarcoma. To validate the diagnosis of UB cancer, the diagnostic and therapeutic procedure codes for each patient with UB cancer were subsequently assessed: 57.49, other transurethral excision or destruction of lesion or tissue of the UB/endoscopic resection of a UB lesion; 57.33, transurethral biopsy of the UB; 57.33, closed (transurethral) biopsy of the UB; 57.7, total cystectomy; 57.6, partial cystectomy; 57.71, radical cystectomy; 60, operations on the prostate and seminal vesicles; 57.32, other cystoscopy; 57.31, cystoscopy through an artificial stoma; and 96.49, instillation of medication into the urinary tract (Supplementary Table 1, available online).

### Follow-up

Because of the coverage provided by the National Health Insurance, all patients were completely followed up till the occurrence of incident UB cancer or death or till the last day of 2013 (up to 12 years of follow-up). Study subjects were not allowed to cross over during the study (Figure 1).

### Statistical analysis

Propensity analysis attempts to affect a balance in observed covariates between studied groups (21). SPSS software with the PSM plug-in allows the estimation of propensity scores by using logistic regression and specifying nearest-neighbor matching. For categorical data, the chi-square test was used to derive *P* values, whereas, for numerical data, the two-sample *t* test was used. The standardized mean difference before and after PSM was calculated. We used a nearest-neighbor algorithm to create groups of hypertensive versus non-hypertensive patients that would be statistically similar to each other and then compared outcomes between these groups of well-matched patients. The outcome was incident UB cancer, which was validated with procedural codes. Person-time data were calculated for each patient. Incidence rates per 10,000 person-years stratified by different variables were calculated. We constructed a multivariable Cox model to derive adjusted hazard ratios (aHRs) and 95% confidence intervals (CIs), after controlling for smoking-related diagnoses, morbid obesity, and chronic liver disease. Cumulative incidence curves were produced using the Kaplan–Meier method and compared using a log-rank test. We performed sensitivity analyses to examine the differential time lag in the risk of UB cancer. Two-sided *P* values of <0.05 were considered statistically significant.

## Results

During a total follow-up duration of 380,525 and 372,020 person-years at risk for the hypertension and non-hypertension groups, 248 and 186 patients developed UB cancer, respectively, representing a 32% increase in the risk (aHR, 1.32; 95% CI, 1.09–1.60). Hypertensive women harbored a statistically significant risk of UB cancer (aHR, 1.55; 95% CI, 1.12–2.13) compared with non-hypertensive women, whereas men with hypertension had an aHR of 1.22 (95% CI, 0.96–1.55), a similar magnitude as the overall risk but not statistically significant on the *P* < 0.05 level (Table 2).

**Table 2.** Incidence of urinary bladder cancer, person-time rate, and stratified analysis showing hazard ratios according to Cox proportional hazards regression in the propensity score-matched study groups.

	Study group	UB cancer	PYs	Rate	Crude HR (95% CI)	Adjusted HR (95% CI)
Total	Non-hypertension (*n* = 39,618)	186	372,020	5.00	1 (reference)	1 (reference)
	Hypertension (*n* = 39,618)	248	380,525	6.52	1.30 (1.08–1.58)*	1.32 (1.09–1.60)*
Men	Non-hypertension (*n* = 20,400)	125	177,509	7.04	1 (reference)	1 (reference)
	Hypertension (*n* = 20,400)	151	179,071	8.43	1.20 (0.95–1.52)	1.22 (0.96–1.55)
Women	Non-hypertension (*n* = 19,218)	61	194,510	3.14	1 (reference)	1 (reference)
	Hypertension (*n* = 19,218)	97	201,454	4.81	1.54 (1.11–2.11)*	1.55 (1.12–2.13)*

Adjusted HR was derived from the multivariable analysis adjusted for medical comorbidities of smoking-related diagnoses, morbid obesity, and chronic liver disease.

*P < 0.01.

CI: confidence interval; PYs: person-years; Rate: incidence rate, per 10,000 person-years.

After PSM, the median age of the entire cohort was 56 years, and there were slightly more male patients than female patients (51.5% versus 48.5%). Approximately 29% and 15% of the patients had diabetes mellitus and gout, respectively. There were significantly more smokers (11.7% versus 11.1%; *P* = 0.004), more obese patients (1.4% versus 0.5%), and more chronic liver disease patients (19.5% versus 14.5%) in the hypertension group than in the non-hypertension group (Table 1).

**Figure 2.**
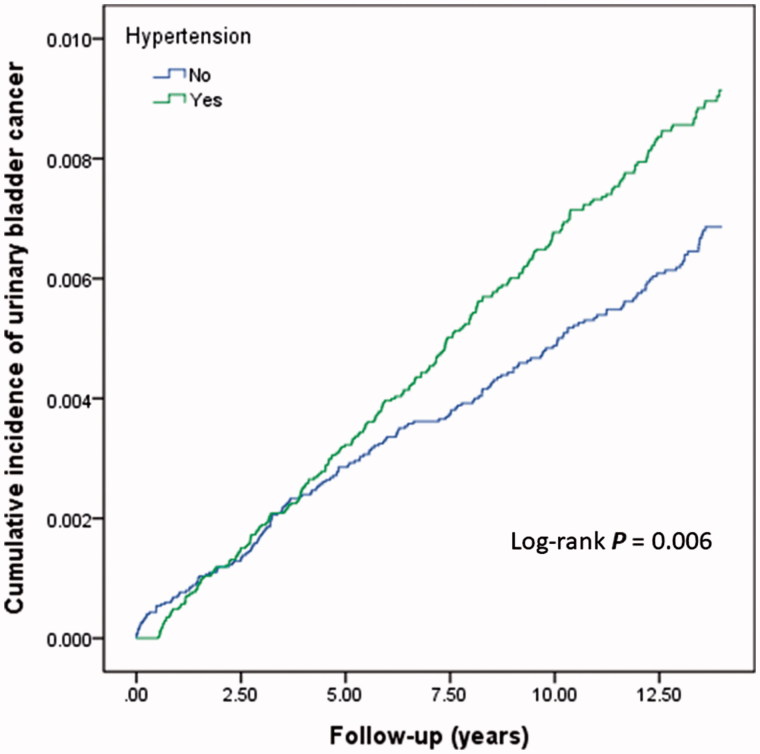
Cumulative incidence of urinary bladder cancer for patients with and without hypertension using the Kaplan–Meier method. Log-rank test was used to compare the curves.

Men (8.43 per 10,000 person-years) had a higher incidence rate of UB cancer than did women (4.81 per 10,000 person-years) (Table 2). The incidence of UB cancer was higher in the hypertension group for either sex. However, in the Cox proportional hazard model with or without controlling for smoking-related diagnoses, morbid obesity, and chronic liver disease, men with hypertension had a statistically insignificant increase in risk (Table 2). Figure 2 shows the cumulative incidence of UB cancer among patients with or without hypertension, using the Kaplan–Meier method. The log-rank test was used to compare the curves (*P* = 0.006), and the Cox model showed a statistically significant higher risk of UB cancer in the hypertension group than in the comparison group.

Results of the sensitivity analyses performed for the entire cohort and different genders stratified by the differential lag time of follow-up are presented in Table 3. Even in the seventh year of follow-up and beyond, there was still a nearly 50% increase in the risk of UB cancer (aHR, 1.46; 95% CI, 1.09–1.97). The risk of UB cancer was significantly increased among men in the hypertension group compared with the non-hypertension group during the first and third to sixth years of follow-up (Table 4).

**Table 3.** Sensitivity analysis showing the effect of differential time lag on the risk of urinary bladder cancer compared between propensity score-matched non-hypertensive and hypertensive groups.

Follow-up	Number of patients (hypertensive/non-hypertensive)	Incidence of UB cancer in (hypertensives/non-hypertensives)	Crude HR (95% CI)	Adjusted HR (95% CI)
Start	39,618/39,618	0/0		
1st year	35,636/34,967	27/18	1.41 (1.15–1.73)***	1.43 (1.17–1.75)***
2nd year	32,541/31,667	24/16	1.41 (1.14–1.74)**	1.42 (1.15–1.76)**
3rd year	30,833/29,829	22/16	1.42 (1.13–1.77)**	1.42 (1.13–1.78)**
4th year	29,948/28,977	19/20	1.51 (1.18–1.92)***	1.51 (1.19–1.93)***
5th year	29,108/28,165	21/13	1.50 (1.16–1.95)**	1.51 (1.16–1.96)**
6th year	28,204/27,365	21/14	1.51 (1.14–2.00)**	1.52 (1.14–2.01)**
7th and beyond	27,368/26,631	123/80	1.45 (1.08–1.96)*	1.46 (1.09–1.97)*

Adjusted HR was derived from the multivariable Cox analysis including confounding variables of smoking-related diagnoses, morbid obesity, and chronic liver disease.

*P < 0.05; **P < 0.01; ***P < 0.001.

**Table 4.** Sensitivity analysis showing the effect of differential time lag on the risk of urinary bladder cancer among male patients in propensity score-matched non-hypertensive and hypertensive groups.

Follow-up	Number of patients (hypertensive/non-hypertensive)	Incidence of UB cancer in (hypertensives/non-hypertensives)	Crude HR (95% CI)	Adjusted HR (95% CI)
Start	20,400/20,400	0/0		
1st year	17,965/17,713	11/19	1.31 (1.02–1.69)*	1.33 (1.03–1.71)*
2nd year	16,145/15,818	16/9	1.27 (0.97–1.66)	1.29 (0.99–1.68)
3rd year	15,085/14,751	11/13	1.34 (1.01–1.77)*	1.35 (1.02–1.79)*
4th year	14,522/14,203	12/13	1.42 (1.05–1.92)*	1.42 (1.05–1.93)*
5th year	13,974/13,666	11/12	1.53 (1.10–2.12)*	1.53 (1.10–2.13)*
6th year	13,370/13,151	14/8	1.50 (1.05–2.13)*	1.50 (1.05–2.14)*
7th and beyond	12,823/12,644	76/51	1.40 (0.97–2.03)	1.41 (0.97–2.04)

Adjusted HR was derived from the multivariable Cox analysis controlling for smoking-related diagnoses, morbid obesity, and chronic liver disease.

*P < 0.05.

## Discussion

To the best of our knowledge, this is one of the very few population-based prospective cohort studies to support the association between essential hypertension and subsequent UB cancer occurrence, a topic that has not been extensively studied yet. This study population was very large, with 39,618 individuals in each arm who were well-matched based on propensity scores for age, gender, and several potential confounding variables. Furthermore, the follow-up period (up to 13 years) was long enough to observe the outcome. Our results demonstrated that more incident UB cancer cases, 248 (0.63% of the hypertension group) versus 186 (0.47% of the comparison group), were found in the hypertension group. The Cox model compared the risk of incident UB cancer between the hypertension and non-hypertension groups, after controlling for smoking-related diagnoses, morbid obesity, and chronic liver disease, and revealed a 32% increase in the risk in the hypertension group. PSM allowed us to seek an adequate balance between the hypertension and non-hypertension groups regarding aspects such as advancing age, excessive alcohol consumption (22), diabetes (23), chronic cystitis (24), and spinal cord injury (25), which are established risk factors for UB cancer. In our propensity analysis, essential hypertension was independently associated with the risk of developing UB cancer during follow-up, after adjusting for smoking-related diagnosis, morbid obesity (26), and chronic liver disease (27).

We demonstrated a 1.5-fold increase in the risk of UB cancer among hypertensive women in this well-matched and controlled cohort study. Our sensitivity analysis for examining how the risk differed during different periods of follow-up demonstrated that the risk (represented as aHRs) was sustained throughout the follow-up period. This study finding is intriguing. A recently published Italian hospital-based case-control study identified that there was a neutral risk (adjusted odds ratio, 0.99; 95% CI, 0.75–1.31) between drug-treated hypertension and urothelial carcinoma of UB (26). Nevertheless, a European cohort study involving 578,700 individuals in Norway, Austria, and Sweden revealed that hypertension was modestly associated with an increased risk of bladder cancer among men (adjusted relative risk, aRR, 1.13; 95% CI, 1.03–1.25) but not in women (aRR, 0.87; 95% CI, 0.69–1.09) (7). In contrast to our study in which women participants had a median age of 56, 74.6% of the women participants in the European cohorts were under 50 years of age, indicating a mostly different population. Why the risk is statistically significant only in women in our study remains to be determined in future research.

The strengths of this study, other than the large number of participants, the prospective cohort study design, and the use of propensity score analysis, are as follows: 1) validation of incident UB cancer performed by checking the procedure codes associated with the diagnosis and management of UB cancer; 2) complete follow-up with no drop-out due to the nature of the universal pan-country coverage of the medical expense for all the insured; 3) absence of recall bias as in the questionnaire research design; and 4) an adequate length of follow-up. Similar to any claims-based cohort studies, there are some potential limitations that should be addressed and cautioned against when analyzing our results. This study did not consider the impact of the risk from antihypertensive drugs. Nevertheless, a recent population-based study using a similar dataset found no impact from antihypertensive use on the risk of UB cancer (6). Their Cox model controlling for age, sex, urbanization level, occupation, income, diabetes, dyslipidemia, stroke, coronary heart disease, chronic obstructive pulmonary disease, alcoholism, and alcoholic liver damage derived a neutral risk (adjusted HR = 1.19; 95% CI, 0.80–1.77) in patients receiving antihypertensive agents versus those who were not. The data of environmental and occupational chemical carcinogen exposure and other personal lifestyle factors, such as fruit and vegetable consumption and physical activity, for each participating individual were not available in the dataset. We cannot fully rule out that the unavailability of this information may have affected the Cox model effect estimates, thus a possible remaining confounding may be present. Although data on smoking habits were not available, we were able to pick up smoking-related diagnoses such as chronic obstructive pulmonary disease and control them in the Cox models.

The broader implications of the findings are as follows: this study supports a positive association between essential hypertension and subsequent UB cancer development, indicating that further studies on the mechanisms underlying the increased risk are warranted. Although the risk in patients with hypertension exists, the clinical relevance of a hazard ratio in the magnitude of 1.32 as a risk estimate from a prevalent exposure representing a 32% increase in the risk of UB cancer is considered modest.
